# Depression and hippocampal subfield volume in older adults

**DOI:** 10.1038/s41398-026-04223-y

**Published:** 2026-07-14

**Authors:** Danielle Luu, Alicia M. Twisselmann, Victoria R. Tennant, Brandon J. Hall, Arjun Mahajan, Audrey Kim, Jamie Terner, Marylan L. Davison, Koral Wheeler, Christopher R. K. Ching, James Hall, Matthew T. Borzage, Leigh Johnson, Arthur W. Toga, Sid E. O’Bryant, Kristine Yaffe, Meredith N. Braskie

**Affiliations:** 1 Stevens Neuroimaging and Informatics Institute, Keck School of Medicine, University of Southern California, Los Angeles, CA USA; 2 University of North Texas Health Science Center, Fort Worth, TX USA; 3 Fetal and Neonatal Institute, Division of Neonatology, Children’s Hospital Los Angeles, Department of Pediatrics, Keck School of Medicine, University of Southern California, Los Angeles, CA USA; 4 Alfred E. Mann Department of Biomedical Engineering, Viterbi School of Engineering, University of Southern California, Los Angeles, CA USA; 5 Department of Regulatory and Quality Sciences, Alfred E. Mann School of Pharmacy and Pharmaceutical Sciences, University of Southern California, Los Angeles, CA USA; 6 UCSF Weill Institute for Neurosciences, San Francisco, CA USA; 7 Keck School of Medicine, University of Southern California, Los Angeles, CA USA; 8 The University of Texas Health Science Center at San Antonio, San Antonio, TX USA; 9 The University of Texas Southwestern Medical Center, Dallas, TX USA; 10 University of Wisconsin School of Medicine and Public Health, Madison, WI USA; 11 Columbia University, New York, NY USA; 12 Fordham University, New York, NY USA; 13 Georgetown University, Washington, D.C. USA; 14 Rush University Medical Center, Chicago, IL USA; 15 University of California Irvine School of Medicine, Irvine, CA USA; 16 University of Pittsburgh, Pittsburgh, PA USA; 17 Wake Forest University School of Medicine, Winston-Salem, NC USA; 18 Washington University, St. Louis, Missouri USA; 19 Midtown Medical Imaging, Fort Worth, TX USA

**Keywords:** Diagnostic markers, Depression

## Abstract

Depression is associated with a higher risk for developing Alzheimer’s Disease (AD) [1], but the mechanisms that underlie the complex relationship between depression and AD remain largely elusive. The hippocampus is a region of the brain that is commonly affected by both AD and depression and serves as a focal point for our study. We aim to understand the relationship of 1) depression and antidepressant medications to hippocampal subfield volume during normal aging, and 2) whether AD risk, as measured by amyloid and tau pathology and *APOE*4 status, impacts these relationships. We studied 2009 ethno-racially diverse cognitively unimpaired older adults aged 50 to 90 years who either had depression (*n* = 630) or were not depressed (*n* = 1379). Participants with depression were further stratified by antidepressant medication usage. High-resolution MRI scans were used to calculate hippocampal subfield volumes that included the CA1, the subiculum, and a composite region that included the CA2, CA3, and the dentate gyrus (CA23DG). Having depression was associated with a smaller CA23DG, independent of amyloid and tau pathology in the brain. Within the subgroup of participants with depression, those who used antidepressant medications had smaller CA1 and CA23DG volumes than those who did not use these medications.

## Introduction

Alzheimer’s Disease (AD) is the most common form of dementia in older adults and is characterized by disruptive cognitive and behavioral deficits. AD is associated with amyloid plaques, tau neurofibrillary tangles, and neuronal loss in the brain [2]. There is a need to study the mechanisms and comorbidities of dementia in the elderly. Depression during one’s lifetime is associated with a higher likelihood of AD later [3]. However, the mechanisms connecting these disorders are unclear. Here, we related depression and associated medications to hippocampal subregional volumes in cognitively unimpaired older adults to better understand 1) how depression and depression medications relate to hippocampal neurodegeneration and 2) whether that neurodegeneration is independent of AD neuropathology.

The hippocampus is one of the earliest brain regions affected by AD [4]. While depression may be an AD risk factor [5–9], its effect on AD-related structural brain changes needs further investigation due to conflicting results. For instance, in depressed AD patients, left hippocampal volume was associated with depression duration in one study (*n* = 262) [10], but in another, depression and hippocampal volume were not associated (*n* = 134), although patients with an early onset depression (age < 60 years) had higher risk for AD [11]. Varying findings may be because of 1) variability in smaller sample sizes and 2) by the time someone manifests the clinical symptoms of AD, their brain has undergone changes that may not be closely related to the initiating factors. By focusing on a large sample (*n* = 2009) of cognitively unimpaired older adults, we evaluated early hippocampal changes related to depression with less variability from unrelated factors associated with cognitive decline and atrophy.

We focus on hippocampal subregions, which are differentially impacted by AD [12] and depression [13, 14]. For instance, CA1 is preferentially affected during early stages of AD [12]. Likewise, CA2, CA3 and the dentate gyrus are targeted by depression in both animal models [15] and humans [13]. Evaluating hippocampal volume as a whole makes it difficult to detect differences in the way disease affects these subregions. Therefore, evaluating hippocampal subregions is more sensitive to AD-like changes than studying the whole hippocampus, allowing better understanding of the relationship between depression and AD. We build upon past work in humans by using a large sample of ethnoracially diverse cognitively unimpaired older adults from the Health and Aging Brain Study - Health Disparities (HABS-HD). Unlike prior studies, we used high resolution MRI of the hippocampus to relate depression to hippocampal volume in three subregions: CA1, subiculum, and a region that includes CA2, CA3, and dentate gyrus (CA23DG). We examined the relationship between depression and CA1, CA23DG, and subiculum volumes in Hispanic/Latino(a) (Hispanic), Black/African American (non-Hispanic Black), and non-Hispanic White older adults. We hypothesized that depression would be associated with smaller volume primarily in CA23DG, consistent with prior work [16, 17] in smaller and less ethnoracially diverse cohorts.

Ethnicity/race and sex are associated with differences in prevalence of depression and AD in the U.S. Major depressive disorder (MDD) and AD are more common in non-Hispanic Black and Hispanic adults than non-Hispanic White adults [18, 19]. In ethnoracially diverse populations, greater depression severity and higher levels of early-life adversity are associated with smaller whole hippocampal volume [20, 21]. Similarly, depression and AD are more common in women than men [22, 23]. Hippocampal volume alterations related to depression also seem stronger in men than women [24, 25]. We hypothesized that the association between depression and lower hippocampal volume would be stronger in males consistent with prior studies. However, many such studies had small samples and did not examine hippocampal subfields. In a sample at least seven-fold larger, and with imaging resolutions at least 36% more detailed than prior studies, we reexamine the relationship between depression and hippocampal subregional volume and evaluate how sex and ethnoracial group affect these relationships.

Carrying the *APOE*4 allele increases the risk for AD pathology [26] and has been associated with increased risk for depression in animal models [27–29], although work in humans did not reflect this [30, 31]. A better understanding of *APOE*4’s interaction with depression on hippocampal volume is needed to develop targeted interventions for brain protection. We evaluated whether *APOE*4 carrier status modifies the relationship between depression and hippocampal subregional volume. Due to the close association of amyloid and tau with neurodegeneration, we also examined whether AD neuropathology, like brain amyloid and tau, plays a role in the association between depression and hippocampal volume. There is a well-documented link between depression and both obesity and physical inactivity, although the relationships are complex and likely bidirectional [32, 33]. Therefore, we examined whether body mass index (BMI) and physical activity levels explained part of the relationship between depression and subfield volume.

Finally, many depressed patients use antidepressant medications, highlighting the importance of analyzing how antidepressant use relates to hippocampal volume. Antidepressant medications may have protective effects against hippocampal atrophy [34, 35]. However, the impacts of antidepressants may differ in older adults. For instance, older women using selective serotonin reuptake inhibitors (SSRIs) had greater cognitive decline after five years than women who were not using antidepressants [36]. We investigated the link between antidepressant use and hippocampal subfield volume in older adults who have depression.

We tested whether antidepressant medication use would be associated with hippocampal subfield volume in depressed people. Antidepressant medication use could be associated with larger subfield volumes in depressed participants due to the reduction in depressive symptoms. Alternatively, antidepressants could be associated with smaller hippocampal volume either due to unknown negative effects on the brain or due to indication bias, where medications are more often prescribed for more severe depression. A better understanding of how depression medications relate to hippocampal structure in older adults can provide crucial insights for clinical decisions.

## Methods

### Participants

We studied 2009 participants from the Health and Aging Brain Study- Health Disparities (HABS-HD). HABS-HD is a multi-ethnic cohort collected at the University of North Texas, Fort Worth using a community-based participatory research approach [37]. Supplementary File 1 lists inclusion and exclusion criteria. Because we studied how depression relates to brain aging, we included participants aged 50–90 years. Sex, race, and ethnicity were all self-reported.

### Ethics approval and consent to participate

All participants undergo an informed consent process, and all processes follow Institutional Review Board protocols of the University of North Texas Health Science Center (registration number: 2016-128). All methods were performed in accordance with the relevant guidelines and regulations.

### MRI acquisition

Participants underwent MRI scanning (3 T Siemens Magnetom Skyra - *n* = 1259 or Siemens Magnetom Vida - *n* = 750) to collect T2-weighted high-resolution MRI scans acquired with oblique coronal slices prescribed perpendicular to the long axis of the hippocampus (TR = 8020 ms; TE = 46 ms; 30 contiguous 2 mm slices; 0.4 × 0.4 mm in-plane resolution).

**Fig. 1:**
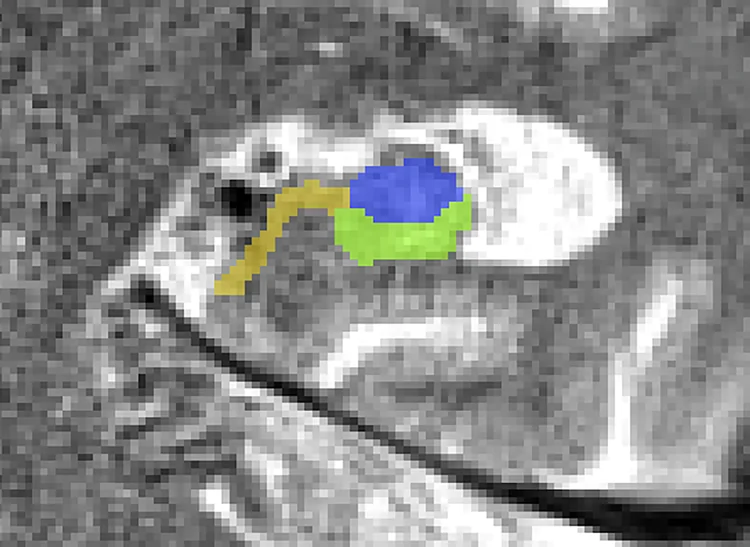
Hippocampal High-Resolution Image. Example of a hippocampal high resolution brain image, showing segmentations of the CA1 (green), CA23DG (blue), and subiculum (yellow).

We used the Automatic Segmentation of Hippocampal Subfields software [38] to segment the MRI scans, and trained reviewers performed manual quality control of segmentation. To optimize reliability of quality control pass-fails and retain the most data during scan quality checks, we grouped subregions into CA1, subiculum, and CA23DG (Fig. 1). This protocol ensured high intra- and inter-rater reliability (Protocol in Supplementary File 2). Only regions that passed quality checks bilaterally for a participant were included in the analyses, resulting in the inclusion of 1908 CA1, 1668 subiculum, and 1896 CA23DG. We used FSL 5.0.9 [39] to extract the volumes and to determine the number of segmented slices from all regions that passed the review. All hippocampal subregional volumes were averaged bilaterally. Intracranial volumes (ICV) were obtained using HippoDeep software [40].

### PET acquisition

We used PET scans to evaluate levels of brain amyloid (^18^F-florbetaben) and tau (^18^F-PI-2620). Amyloid PET scans were collected using a Siemens MCT 20 PET/CT or Siemens Biograph Vision 450 PET/CT scanner. Tau PET scans were collected using a Siemens Biograph Vision 450 PET/CT scanner. Amyloid PET scans were processed using the ADNI3 protocol [41], and standardized uptake value ratios (SUVRs) were calculated using the whole cerebellum as the reference region. We calculated global amyloid as the mean amyloid PET SUVR of the frontal, cingulate, lateral parietal, and lateral temporal lobes. We calculated medial temporal (entorhinal cortex, hippocampus, and amygdala) tau PET SUVR using the inferior gray matter of the cerebellum as the reference region consistent with prior work [42]. All regions of interest were defined using T1-weighted volume scans (MPRAGE) and FreeSurfer v5.0.9.

### Diagnostic classifications

Participants were diagnosed with mild cognitive impairment (MCI) and dementia based on standard criteria [37]. They were diagnosed as cognitively unimpaired if they failed to meet the requirements for MCI or dementia (Supplementary File 1). Participants with no cognitive complaints or functional decline who had a low performance on a single neuropsychological test were diagnosed with normal cognition (Supplementary Table 1).

Participants were measured on the Geriatric Depression Scale (GDS) with subscales (long form). The GDS has a mean sensitivity of 0.753 and a mean specificity of 0.770 of detecting depression in the elderly compared with gold standard external case criteria [43]. Participants were classified as having depression if they had a GDS score ≥ 10 (regardless of depression history) [44] or if they had a history of depression. History of depression is self-reported and defined as having a depression diagnosis from a healthcare provider. Participants were categorized as non-depressed if they had a GDS score ≤ 9 and did not have a history of depression (Table 1).

**Table 1:** Demographics by Depression Status (mean ± SD).

	Depressed	Non-Depressed
N	630	1379
Sex: n (%)*		
Male	140 (22.2%)	528 (38.3%)
Female	490 (77.8%)	851 (61.7%)
Ethnoracial group: n (%)*		
Hispanic	260 (41.2%)	503 (36.5%)
Non-Hispanic Black	105 (16.7%)	283 (20.5%)
Non-Hispanic White	265 (42.1%)	593 (43.0%)
*APOE*4 positive: n (%)	135 (21.4%)	304 (22.0%)
15.53% missingness		
Mean age (years)*	63.72 ± 8.15	65.14 ± 8.50
Mean education (years)*	12.73 ± 4.55	13.87 ± 4.19
Mean BMI*	31.17 ± 6.72	30.22 ± 6.52
0.80% missingness		
Mean Rapid Assessment of Physical Activity*	4.11 ± 1.55	4.61 ± 1.60
0.10% missingness		
Mean Global Amyloid SUVR	1.02 ± 0.12	1.02 ± 0.12
63.17% missingness		
Mean MTL Tau SUVR	1.11 ± 0.11	1.12 ± 0.11
79.09% missingness		

The demographic breakdown of the sample population by depression status. Standard deviations between groups are similar. Pearson’s chi-squared tests of independence were performed to assess the relationship between depression status and sex, ethnoracial group, and *APOE*4 positivity. Student’s t-tests were performed to assess the relationship between depression status and age, education, BMI, RAPA, global amyloid SUVR, and medial temporal lobe tau SUVR. Significant relationships (*p* < 0.05) are marked with *. *APOE* genotype was missing in 312 participants. Power analysis was performed to ensure power of > 0.80 in this sample.

Within the depressed group, participants who had self-reported medication use information were identified as either medicated or non-medicated for depression based on internal medication coding (Table 2). Supplementary Table 2 lists the number of participants taking each type of depression medication and its dose.

**Table 2:** Demographics for Depressed Participants by Medication Usage (mean ± SD).

	Medicated	Non-Medicated
N	263	367
Sex: n (%)		
Male	56 (21.3%)	84 (22.9%)
Female	207 (78.7%)	283 (77.1%)
Ethnoracial group: n (%) *		
Hispanic	68 (25.9%)	192 (52.3%)
Non-Hispanic Black	43 (16.3%)	62 (16.9%)
Non-Hispanic White	152 (57.8%)	113 (30.8%)
Mean age (years)	64.3 ± 7.8	63.3 ± 8.4
Mean education (years) *	14.1 ± 3.7	11.8 ± 4.8
Geriatric Depression Scale score (points) *	8.9 ± 6.7	11.0 ± 6.5
Mean BMI *	32.1 ± 7.1	30.5 ± 6.4
1.0% missingness		
Mean Rapid Assessment of Physical Activity	4.1 ± 1.5	4.1 ± 1.6
0.2% missingness		

The demographic breakdown of the sample population by medication usage. Standard deviations between groups are similar. Pearson’s chi-squared tests of independence were performed to assess the relationship between medication status and sex and ethnoracial group. Student’s t-tests were performed to assess the relationship between medication status and age, education, GDS score, BMI, and RAPA. A higher GDS score indicates greater levels of depression. Significant relationships (*p* < 0.05) are marked with *. The non-Hispanic White sample had the highest percentage of depressed participants who used antidepressant medications. Power analysis was performed to ensure power of > 0.80 in this sample.

Aripiprazole represents an atypical antidepressant treatment used for those who are resistant to more typical antidepressants [45]. We performed a sensitivity analysis to evaluate whether aripiprazole was driving medication effects in the hippocampus (Supplementary Table 3). Supplementary Table 4 shows medication characterization by drug classes.

### Obesity and activity levels

Depression and depression severity are associated with obesity and low levels of physical activity. We used BMI, calculated as (weight in pounds)/(height^2^ in inches) × 703, to measure obesity. We used the Rapid Assessment of Physical Activity (RAPA) to assess the frequency and intensity of physical activity. The questionnaire consists of 7 questions regarding the level and intensity of physical activity in which respondents typically engage. The RAPA has a sensitivity of 0.81 for assessing physical activity levels in adults compared with the longer, previously-validated Community Healthy Activities Model Program for Seniors (CHAMPS) questionnaire [46].

### Demographics

We included 2009 cognitively unimpaired participants between the ages of 50–90 (mean 64.69 ± 8.41) years who had hippocampal volume data for at least one subfield at baseline. All MRI scans were acquired within one year of the participants’ clinical interview dates. Demographic information is split by depression status (Table 1) and medication status (Table 2).

### *APOE*4 genotyping

*APOE*4 genotyping was conducted using commercially available TaqMan genotyping kits as described previously [47]. *APOE*4 positivity was defined by the presence of at least one *APOE*4 allele.

### Statistical analyses

Statistical tests were performed in R studio 2024.12.0 + 467, using ggplot, dplyr, and MASS packages. All continuous variables were checked for normality via histograms and converted to z-scores. Depression was evaluated using two overlapping but different measures: (1) clinical depression diagnosis based on prior history or GDS ≥ 10, and (2) continuous Geriatric Depression Scale (GDS) scores. We used false discovery rate (FDR) correction to correct for multiple comparisons across three brain regions separately within each depression predictor.

### Diagnostic group differences

We assessed differences in sex, ethnicity, *APOE*4 positivity, age, education, BMI, RAPA, global amyloid SUVR, and medial temporal tau SUVR between depressed and non-depressed participants (Table 1). We used Pearson’s chi-square tests of independence to compare categorical variables between depression status groups and Welch’s independent two-sample t-tests to compare continuous variables. Similarly, differences between sex, ethnicity, age, education, GDS score, BMI, and RAPA were assessed for medication status groups (Table 1).

### Depression and subregional volumes

#### Base model

We ran separate multiple linear regressions to assess whether depression was associated with hippocampal subfield volume in the mean bilateral CA1, CA23DG, and subiculum. We also assessed whether total GDS score (a standardized, continuous variable) and age of onset of depression were associated with these regions. Covariates included age, sex, years of education, number of MRI slices segmented, ICV, and scanner.

#### Model 2: Base model with BMI and RAPA

Higher BMI is associated with more depressive symptoms in adults [48]. Depression is also linked to physical inactivity, where exercise can play a role in alleviating depressive symptoms [49]. Therefore, for Model 2, we added BMI and RAPA as covariates to the linear regressions that were significant in our base model. We evaluated whether either variable explained the relationships between depression and hippocampal subfield volumes.

As a supplementary analysis, we included a model that added self-reported ethnoracial group as a covariate to Model 2 to determine whether it helped explain the relationship between depression and hippocampal volumes. We use this variable as a proxy for factors related to ethnoracial group - like cultural norms, systemic and interpersonal racism, comorbidities, and socioeconomic status - that may impact depression. Because this is a proxy measure, it must be interpreted with caution as there may be unmeasured differences in group composition across ethnicity or differences related to other factors.

#### Model 3: Base model with BMI, RAPA, and Amyloid/Tau/*APOE*

Depression may be a risk factor for developing AD but alternatively may be a symptom of preclinical accumulation of amyloid or tau. To investigate whether amyloid and tau pathology helps explain the association between depression and hippocampal subfield volume, in Model 3, we performed separate multiple linear regressions between depression and hippocampal subregion volume, including all covariates in Model 2 plus global amyloid, medial temporal lobe tau, or *APOE*4 positivity. Because we had these measures in different subsamples of participants, we added each variable separately to not limit our sample size excessively. We performed this analysis in a subset of participants who had available measures for global amyloid (*n* = 684), medial temporal lobe tau (*n* = 386), and *APOE*4 status (*n* = 1587).

#### Model 4: Base model with BMI, RAPA, and medications

In Model 4, we included the covariates listed in Model 2 and added medication use. This allowed us to evaluate whether the significant relationship between depression and hippocampal subregion volume in the full sample was related to current antidepressant use rather than to diagnosis. The correlation between depression and medication use was R = 0.39 - low enough that including these covariates in the same model would be unlikely to violate the assumptions of the test.

#### Comparing history of depression and GDS score

To compare the separate effects of depression history and GDS score, we stratified the sample by 1) participants who had GDS ≥ 10 regardless of depression history, and 2) participants who had a history of depression but controlled - with GDS < 10. We tested the relationship between GDS score and CA23DG volume, as well as between medication status and CA23DG volume in these two groups.

### Medication status and subregional volumes

We ran separate multiple linear regressions only in participants with depression to examine the relationship between medication status and hippocampal subfield volume in all three regions. We first ran regressions using the same covariates as in our base model above: age, sex, years of education, number of MRI slices segmented, ICV, and scanner. For regions where we saw a significant association with medication status in the base model, we ran another set of regressions with additional covariates, BMI and RAPA (Model 2).

### Interactions by *APOE*4, sex, and ethnoracial group

We further assessed the above models by testing the interactive effects of *APOE*4 positivity, sex, and ethnoracial group with depression within each hippocampal subregion. All interaction analyses included covariates from Model 2.

As an exploratory study, we ran linear regression models separately in subgroups divided by *APOE*4 positivity (*APOE*4+ or *APOE*4-), sex (male or female), and ethnoracial group (Hispanic, non-Hispanic Black, or non-Hispanic White).

## Results

### Diagnostic group differences

Between the depressed and non-depressed groups (Table 1), there were significant differences in sex (χ^2^ = 49.57, *p* < 0.001, greater proportion of depressed females), racial/ethnic group (χ^2^ = 6.03, *p* = 0.049, greater proportion of depressed Hispanic participants), age (t = 3.58, *p* < 0.001, non-depressed people older), years of education (t = 5.33, *p* < 0.001, non-depressed people more educated), BMI (t = −2.95, *p* = 0.003, higher BMI in depressed people), and RAPA (t = 6.59, *p* < 0.001, higher levels in non-depressed people). There were no significant differences in depression by *APOE*4 positivity (χ^2^ = 0.12, *p* = 0.73), global amyloid SUVR (t = 0.12, *p* = 0.91), or medial temporal tau SUVR (t = 1.56, *p* = 0.12). Between the medicated and non-medicated depressed participants (Table 2), there were significant differences in ethnoracial group (χ^2^ = 52.58, *p* < 0.001, non-Hispanic White participants most likely and Hispanic participants least likely to be medicated for depression), years of education (t = 6.88, *p* < 0.001, medicated people more educated), GDS score (t = −3.89, *p* < 0.001, lower scores in medicated participants), and BMI (t = 2.84, *p* = 0.005, higher BMI in medicated participants). There were no significant differences in sex (χ^2^ = 0.14, *p* = 0.71), age (t = 1.46, *p* = 0.15), or RAPA (t = −0.58, *p* = 0.56) on medication status in depressed participants.

### Depression and subregional volumes

#### Base model

**Fig. 2:**
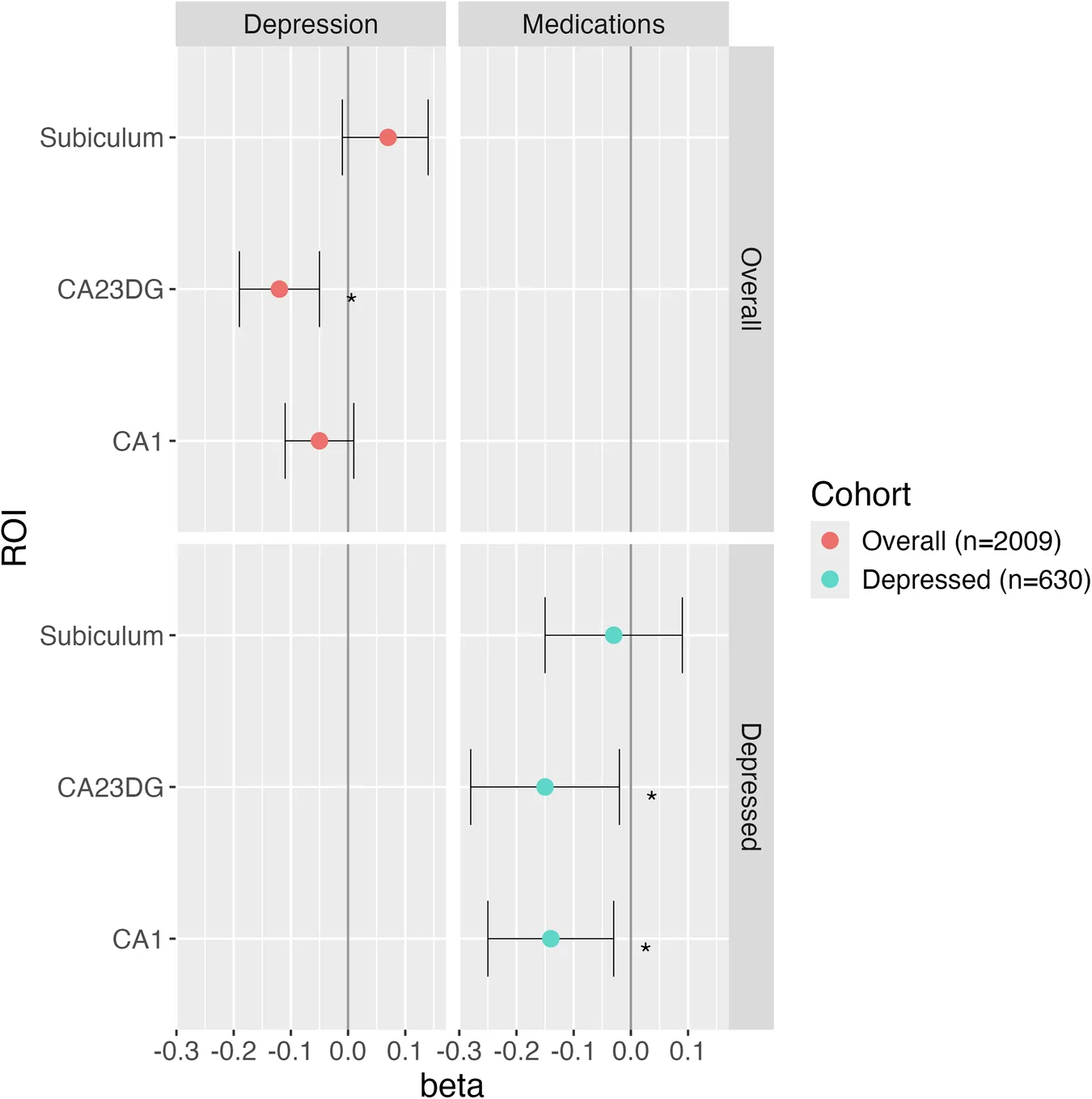
Forest Plot of Base Linear Model. Beta values of linear regressions for the base model, with age, sex, years of education, number of MRI slices segmented, ICV, and scanner as covariates. *P*-values that were significant after FDR correction are marked with *.

Having depression was associated with a smaller CA23DG (β = −0.12, *p* = 0.005). Depression was not significantly associated with CA1 (β = −0.05, *p* = 0.13) or subiculum volumes (β = 0.07, *p* = 0.12) (Table 3). Beta values are plotted in Fig. 2.

**Table 3:** Base Linear Regression Model.

			CA1		CA23DG		Subiculum	
Subgroup	n	Predictor	β	p	β	p	β	p
Overall	2009	Depression	−0.05	0.13	−0.12	0.005*	0.07	0.12
Depressed	630	Medication	−0.14	0.03*	−0.15	0.03*	−0.03	0.63

Separate linear regressions were run to test the effect of depression on hippocampal subfield volumes in the overall sample. Separate linear regressions were run to test the effect of antidepressant medication use on hippocampal subfield volumes in the depressed sample. Covariates included age, sex, years of education, number of MRI slices segmented, ICV, and scanner. All *P*-values listed are FDR-corrected.

GDS and age of onset of depression were not significantly associated with any hippocampal subfield volumes. (Supplementary Table 5)

#### Model 2: Base model with BMI and RAPA

With BMI and RAPA as additional covariates, depression remained associated with a smaller CA23DG (β = −0.11, *p* = 0.01). (Supplementary Table 6)

See Supplementary Table 7 for results after ethnoracial group was added as a covariate.

#### Model 3: Base model with BMI, RAPA, and Amyloid/Tau/*APOE*

With global amyloid added as a covariate to Model 2 (*n* = 684), depression and CA23DG volume were no longer significantly associated (β = −0.10, *p* = 0.11). However, the β was comparable to Model 2 (β = −0.11), indicating the change in *p* value likely relates to the smaller sample size rather than a change in effect. With medial temporal lobe tau added as a covariate to Model 2 (*n* = 386), the relationship between depression and CA23DG volume was significant (β = −0.17, *p* = 0.04).

With *APOE*4 positivity added as a covariate to Model 2 (*n* = 1587), the association between depression and CA23DG remained significant (β = −0.11, *p* = 0.01).

#### Model 4: Base model with BMI, RAPA, and medications

With antidepressant medication status added to the model, the association between depression and a smaller CA23DG was diminished (β = −0.07, *p* = 0.11). In this model, antidepressant medication use was associated with smaller CA23DG (β = −0.14, *p* = 0.005).

### Comparing history of depression and GDS in participants

In participants with depression, we evaluated whether extent of current symptoms (GDS score) was more related to smaller CA23DG in those with current uncontrolled symptoms (GDS ≥ 10) or with history of diagnosis but controlled symptoms (GDS < 10). We included the covariates outlined in Model 2 (Base Model with BMI and RAPA). In the same participant groups but a separate analysis, we related antidepressant medication use to CA23DG volume. In participants with GDS ≥ 10 (without considering prior depression diagnosis) (*n* = 343), there was no association between either GDS score (β = 0.002, *p* = 0.97) or medication status (β = −0.02, *p* = 0.84) and CA23DG volume. In participants with a history of depression but a GDS < 10 (*n* = 287), GDS score was associated with smaller CA23DG volume at a trend level (β = −0.17, *p* = 0.06), and antidepressant medication use was associated with smaller CA23DG volume (β = −0.21, *p* = 0.02) (Supplementary Table 8).

### Interactions by *APOE*4, sex, and ethnoracial group

Interactions were run on Model 2 (Base model with BMI and RAPA as covariates). A formal interaction of *APOE*4 positivity and depression did not yield significant results on CA1, CA23DG, or subiculum volumes. There were trend level interactions between sex and depression in the CA1, CA23DG, and subiculum. Lastly, there were no significant interactions between ethnicity and depression in any of the subregions (Table 4).

**Table 4:** Interactions by Sex, *APOE*4 Positivity, and Ethnicity.

	CA1		CA23DG		Subiculum	
	β	p	β	p	β	p
Sex	0.13	**0.09**	0.16	**0.09**	0.14	**0.09**
*APOE*4 Positivity	0.004	0.96	0.11	0.70	0.03	0.96
Ethnicity	−0.07	0.97	−0.01	0.97	−0.12	0.97

Formal interactions were run for sex, *APOE*4 positivity, and ethnicity and depression on CA1, CA23DG, and subiculum volumes. Covariates included age, sex, years of education, number of MRI slices segmented, ICV, scanner, BMI, and RAPA. All *P*-values listed are FDR-corrected.

Bolded *p* values are at trend-level significance (*p* < 0.10).

Linear regression models were run in stratified groups (*APOE*4 positivity, sex, and ethnicity) as an exploratory analysis. When subset by *APOE*4 status, depression was associated with a smaller CA23DG (β = −0.14, *p* = 0.01) in *APOE*4- but not *APOE*4 + (β = −0.04, *p* = 0.64) participants. Depression was associated with a smaller CA1 (β = −0.16, *p* = 0.02) and smaller CA23DG (β = −0.23, *p* = 0.01) in males but not in females (CA1: β = −0.01, *p* = 0.84, CA23DG: β = −0.08, *p* = 0.10). There were no significant associations between depression and hippocampal subregion volumes when stratified by ethnoracial group (Supplementary Table 9).

Additionally, we examined the interactions between global amyloid or medial temporal tau PET levels and depression on hippocampal subfield volume. The results were not significant for any region (Supplementary Table 10).

### Medication status and subregional volumes

#### Base model

In participants who had depression, using antidepressant medications was associated with smaller CA1 (β = −0.14, *p* = 0.03) and smaller CA23DG (β = −0.15, *p* = 0.03) (Table 3).

#### Base model with BMI and RAPA

With BMI and RAPA variables added, antidepressant medication use was associated with smaller CA1 (β = −0.12, *p* = 0.06) and smaller CA23DG (β = −0.12, *p* = 0.07) at trend levels (Supplementary Table 6).

## Discussion

### Depression and hippocampal subfield regions

Depression was associated with a smaller CA23DG but not CA1 or subiculum, consistent with prior work showing smaller hippocampal subfield volume, specifically in the dentate gyrus [16] and CA1-3 [17]. Depression was not associated with subiculum volume in our analyses. This is supported by previous work showing stronger effects of depression in CA1-4 without the subiculum [50]. The core substructures that make up the trisynaptic circuit of the hippocampus may be more closely related to depression than the subiculum is. GDS score was not associated with any subfield volumes in the full sample, possibly because GDS scores typically decrease with antidepressant medication treatment, while the relationship between depression and hippocampal structure may relate more to a history of depression.

When comparing participants with GDS ≥ 10 (indicating current symptoms) and participants with a history of depression but GDS < 10, we found GDS scores and medication use were associated with smaller CA23DG volume only in the latter group. Almost half of participants with GDS ≥ 10 did not have a self-reported depression diagnosis, indicating they were newly diagnosed and weren’t aware of their own diagnosis, possibly because the depression was less severe. In contrast, the group with GDS < 10 was aware of their depression diagnosis, indicating that although their current symptoms were controlled, their depression had been severe enough to warrant a diagnosis previously and may have longer duration overall on average than in the GDS ≥ 10 group. Our results suggest a longer duration of depression may be more important to hippocampal health than current symptom severity. Alternatively, those with GDS < 10 and history of depression are more likely to be medicated for depression, thus lowering their GDS scores. It is possible that medications themselves are driving the association with hippocampal structure. Perhaps the effects we see are related more to medication use than longer duration of depression.

### Antidepressant medications

Depressed individuals who used antidepressant medications had a smaller CA1 and CA23DG volume. Our work is consistent with that of the ENIGMA MDD working group, which found patients with MDD who had a higher percentage of antidepressant use had a trend-wise lower hippocampal volume [51] compared to those with lower levels of antidepressant use. This appears counterintuitive, since medications should work to alleviate the harmful physiological effects of depression in the long term, therefore acting as a protective factor against further hippocampal damage [52]. Perhaps those with more severe depression are more likely to be prescribed depression medications than those who have milder depressive symptoms [53]. It is possible that the relationship between medications and smaller CA1 and CA23DG can be attributed to depression effects. We found GDS score was lower in those who used depression medication compared to those who did not, likely indicating the medications were working as prescribed to alleviate depression symptoms. Depression severity can change before and after starting medication, so a longitudinal study that includes pre-medication severity would produce a more holistic picture.

We did not have longitudinal information on the severity of depression. We did investigate whether onset of depression predicted hippocampal subfield volume, but did not find significant results in any region. However, the onset of depression may not always correspond with the onset of depressive symptoms. Duration of depression and medication use would be helpful in separating the effects of medications from those of depression severity, but these measures were not available to us.

Of course, medications may have a negative impact on hippocampal structure. This possibility should be further investigated through randomized, controlled trials.

### The role of *APOE*4, amyloid, and tau

The interaction of *APOE*4 positivity and depression on hippocampal subfield volumes was not significant in any subregion. We did not detect an association between depression and *APOE*4 positivity (Supplementary Table 11), which is consistent with prior work in AD patients and cognitively unimpaired older adults [31]. While formal interaction tests were not significant, stratified analyses (Supplementary Table 9) are provided for reference and may help inform future studies or power calculations.

Although the association between depression and a smaller CA23DG was diminished when controlling for brain amyloid levels, the similar β values indicate this may be due to smaller sample sizes. Amyloid and tau accumulation, as well as the *APOE*4 genotype, can influence hippocampal volume in cognitively unimpaired adults, even before clinical symptoms of AD appear [54–57]. However, even after controlling for these factors, depression was still associated with smaller CA23DG volume. Future studies should include larger samples to clarify the role of AD neuropathology in this relationship.

### Sex differences

The interaction of sex by depression on hippocampal subfield volume was trend-level significant, and stratified analyses suggested somewhat stronger associations between depression and smaller CA1 and CA23DG volumes in males (Supplementary Table 6). However, these effects attenuated after adjusting for ethnicity/race, and given the nonsignificant interactions, we interpret these findings cautiously.

Notably, after adding ethnicity/race to our model, the significance diminishes to a trend level in males in both regions (Supplementary Table 7), suggesting that part of the effect we saw in males may be related to differences in sex distribution among our ethnoracial groups. However, it is possible that other factors contribute to the attenuated effect.

### Limitations

Depression and antidepressant medication use in participants was based on self-reported information rather than medical records, which may not always be accurate. Future work that uses participant medical records to assess depression diagnosis and medication information would be useful for validation of these results. Further, we did not know the time or duration of antidepressant use, so we were unable to distinguish whether medication effects were related to the medications themselves or whether depressed people who were prescribed antidepressants may have had more severe depression on average prior to medication than depressed people who were not prescribed antidepressants. A longitudinal study that includes information on depression and medication duration and premedicated severity would better capture the effects of depression and antidepressants.

To maintain statistical power, we did not perform specific analyses by medication dosage or type. As our sample grows, we will compare types of antidepressant medications, such as SSRIs, serotonin-norepinephrine reuptake inhibitors (SNRIs), and tricyclic antidepressants (TCAs), and examine their separate effects on the hippocampus.

Cognitive behavioral therapy was not considered here, since this data was not available, but may have appreciable impacts on depression too. We did not consider the effects of diet or social interaction levels on depression.

Due to limited sample size, especially when examining amyloid and tau PET data and medications, there is potential for underpowered results. Therefore, we recognize that further studies are needed to draw stronger conclusions regarding the relationship between brain pathology, antidepressants, and depression.

## Conclusions

We found that depression was related to smaller CA23DG volume. Despite the detrimental effects that amyloid and tau pathology and *APOE*4 genotype separately have on the hippocampus, these factors did not modify this relationship. Antidepressant medication use was linked to smaller CA1 and CA23DG, but this may be due to indication bias. It is important to study AD in the context of mental health, since rates of depression rise with age [58]. Moreover, it is crucial to investigate how depression and antidepressant medication use affect the hippocampus, a region of the brain that is heavily impacted by AD. Understanding the relationship between depression and the development of AD is important for exploring preventative measures against brain atrophy and dementia.

## Supplementary information

Supplemental Material

## Data Availability

The data that support the findings of this study are openly available in the Image and Data Archive (IDA) at https://ida.loni.usc.edu/.
