# Supplementary material for: Depression and hippocampal subfield volume in older adults
Source: Transl Psychiatry. 2026 Jul 14;16:540. doi: 10.1038/s41398-026-04223-y (PMC13649707; doi:10.1038/s41398-026-04223-y)
Supplement: Supplementary file 1 [file 41398_2026_4223_MOESM1_ESM.docx]

**Supplementary File 1. HABS-HD Exclusion Criteria and Cognitive Diagnosis**

Inclusion criteria include:

1) Age 30 and above

2) Self-reported race and ethnicity of Hispanic, non-Hispanic White or non-Hispanic Black/African American

3) Willingness to provide a blood sample

4) Willing and capable of undergoing magnetic resonance imaging (MRI) and positron emission tomography (PET) scan of the head

5) Capable of providing a reliable informant

6) Fluent in English or Spanish

Exclusion criteria include:

1) Type 1 diabetes

2) Current uncontrolled inflammatory condition (current urinary tract infection, lupus)

3) Diagnosis of cancer in the last 12 months (exception includes non-melanoma skin cancer or non-metastatic prostate cancer)

4) Undergoing or have had chemotherapy or radiation treatment in the last 12 months

5) Active mental illness that could impact cognitive functioning (exception includes depression or anxiety)

6) Traumatic brain injury (TBI) with loss of consciousness in the last 12 months

7) Current alcohol or substance abuse

8) Severe medical conditions that can impact cognition (current end stage renal failure)

9) Presence of a condition/device that prevents the HABS-HD partner from undergoing an MRI or PET scan

Consensus cognitive diagnosis:

Consensus cognitive diagnosis was determined by a combination of assessments by licensed clinicians, medical history, current medications, and objective measures. Participants were diagnosed with mild cognitive impairment (MCI) if they met all the following criteria: (1) a complaint of cognitive change, (2) a CDR sum of boxes score of 0.5-2.0, and (3) performance ≤ 1.5 standard deviations below z-score adjusted norms on at least one cognitive test. Patients were diagnosed with dementia if they (1) had a CDR sum of boxes score ≥ 2.5 and (2) scored ≥ 2 standard deviations below the mean on two or more cognitive tests. Participants were diagnosed as cognitively unimpaired if they failed to meet the requirements for MCI or dementia. A participant with no cognitive complaints or functional decline who had a low performance on a single neuropsychological test would be diagnosed with normal cognition.

**Supplementary File 2. HABS-HD Hippocampal High Resolution (HHR) Protocol**

**Acquisition:**

Scans were acquired on a Siemens Skyra 3T MRI March 2017-December 8, 2020 or a Siemens Vida 3T MRI starting December 9, 2020. Data summaries indicate the scanner and Vida protocol (Skyra, Vida1, Vida2) used to acquire the data. Scanner and protocol were included as covariates in all statistical analyses.

**Tools Used:**

1. Dicom to Nifti converter: dcm2niiX - version v1.0.20181125: <https://www.nitrc.org/plugins/mwiki/index.php/dcm2nii:MainPage>
2. FSL - version 5.0.7: <https://fsl.fmrib.ox.ac.uk/fsl/oldwiki/>
3. Advanced Normalization Tools (ANTs) - v2.1.0: <https://github.com/ANTsX/ANTs/blob/master/README.md>
4. Automatic Segmentation of Hippocampal Subfields (ASHS) - v1.0.0 (September 2017): <https://sites.google.com/view/ashs-dox/home>

**Reliability testing for all raters:**

Before lab members are approved to quality check hippocampal high resolution scan segmentations in the HABS-HD cohort, they need to complete a training set and take a reliability test using a set of scans that have already been quality checked and normed by a team of experienced raters. The trainee had to reach a Cohen’s kappa of at least 0.6 between their answers and the normed answer key created by multiple expert raters in order to pass the reliability set.

**Preprocessing:**

Before Automatic Segmentation of Hippocampal Subfields (ASHS) was run on a scan we performed the following preprocessing on the MPRAGE (T1-weighted structural scan) that served as input into ASHS along with the T2-weighted hippocampal high-resolution (HHR) scan. 1) We used FSL tool fslreorient2std to reorient the MPRAGE to standard template images (MNI152) space. 2) We used the FSL tool fslroi to crop the reoriented MPRAGE to remove extraneous non-brain matter (for instance neck) that can impair coregistration. 3) We used ANTs N4 Bias Correction tool to perform bias correction on the reoriented and cropped T1-weighted scan.

**Hippocampal High Resolution (HHR) scan processing:**

ASHS software was used to segment the hippocampal subfields and quantify the volume. The required inputs for ASHS are the HHR image and the preprocessed T1-weighted MRI.

**Visual quality check of regions of interest segmentations**

Using FSLview, we loaded the left and right hippocampal subregion segmentation masks overlaid on top of the corresponding participant’s hippocampal high-resolution image.

We determined the first slice of segmentation and erased any segmentation occurring before the first slice. The first slice we included in our segmentation was the one in which the hippocampus went from a double-layer structure to a single-layer structure. Visually, this was the slice in which 1) the hippocampal body was separated completely from the rest of the surrounding gray matter with border cerebrospinal fluid (CSF) and 2) there was a clear medial hypointense boundary tight to the hippocampal body.

We chose our hippocampal subregion delineations to optimize information provided while still allowing for reliable visual checks with a pass/fail decision for each regional segmentation in a large sample across multiple raters. Our regions, using a modified ASHS atlas trained on our HABS-HD data set are shown on the figure below:

o	CA1 (green)

o	CA23DG (blue), which includes CA2, CA3, and dentate gyrus.

o	Subiculum (yellow)

In the coronal view, if there were **4 or more** instances of oversegmentation or undersegmentation of a subregion mask at least as large as a 2 x 2 square of voxels, the segmentation for that subregion was failed.

We determined the last slice and erased any automatic segmentation occurring after the last slice. The last slice was defined as the slice two slices anterior to the slice in which at least two branches of the superior and inferior colliculi cease to be visible. See example below in which the slice labeled as n indicates when the superior and inferior colliculi are not visible, the slice labeled as n-1 indicates where the colliculi are fully formed, and the slice labeled as “keep” is the last slice of segmentation that we keep.

We used fslstats to derive volume values for each passed segmentation generated by ASHS.

We also output the number of slices that were segmented for each scan. To account for differences in positioning and head size, the number of slices and intracranial volume, were both included as covariates in all analyses that included the hippocampal subregional volumes.

All analyses and quality control procedures were performed by our team as a co-lead lab for the HABS-HD Neuroimaging Core. We make these neuroimaging values available to researchers through the Laboratory of Neuroimaging Image & Data Archive (LONI IDA) at the University of Southern California.

**Supplementary Table 1. Mean Cognitive Test Scores**

| **Test** | **Overall** | **Depressed** | **Non-depressed** |
| --- | --- | --- | --- |
| SEVLT Delayed Recall  0.7% missingness | 8.7 ± 2.8 | 8.7 ± 2.8 | 8.7 ± 2.8 |
| Logical Memory  0.05% missingness | 38.3 ± 10.1 | 37.7 ± 10.6 | 38.5 ± 9.9 |
| MMSE * | 28.2 ± 2.1 | 27.9 ± 2.5 | 28.3 ± 2.0 |
| Trail Making Test *  0.2% missingness | 36.9 ± 15.8 | 38.9 ± 18.3 | 36.0 ± 14.4 |

Supplementary Table 1. Mean cognitive test scores for cognitively unimpaired participants (no MCI, no dementia). SEVLT = Spanish English Verbal Learning Test (Delayed Recall), MMSE = Mini Mental State Exam. Student’s t-tests were performed to assess the relationship between depressed and non-depressed participants for each cognitive test. * indicates a p-value < 0.05.

**Supplementary Table 2. List of Medications Coded as Antidepressants and Frequency and Dose of Medications**

| **Generic Name** | **Drug Names** | **Frequency** | **Mean Dose ± SD (mg)** |
| --- | --- | --- | --- |
| aripriprazole | Abilify, Abilify MyCite | 7 | 8.4 ± 6.9 |
| amitriptyline |  | 5 | 35.0 ± 17.3 |
| amtriptyline/chlordiazepoxide |  | - | - |
| amtriptyline/perphenazine |  | - | - |
| amoxapine |  | - | - |
| brexpiprazole | Rexulti | - | - |
| bupropion | Aplenzin, Budeprion SR, Forfivo XL, Wellbutrin SR, Wellbutrin XL | 57 | 190.9 ± 82.7 |
| citalopram | Celexa | 36 | 22.1 ± 9.5 |
| desipramine | Norpramin | - | - |
| desvenlafaxine | Pristiq | 4 | 75.0 ± 28.9 |
| doxepin |  | 3 | 41.7 ± 14.4 |
| duloxetine | Cymbalta, Irenka | 32 | 68.1 ± 53.4 |
| escitalopram | Lexapro | 34 | 16.9 ± 15.4 |
| esketamine | Spravato | - | - |
| fluoxetine | Prozac | 22 | 25.8 ± 15.5 |
| fluoxetine/olanzapine | Symbyax | - | - |
| imipramine | Tofranil | 2 | 17.5 ± 10.6 |
| isocarboxazid | Marplan | - | - |
| l-methylfolate | Deplin, L-Methylfolate Forte, XaQuil XR | - | - |
| levomilnacipran | Fetzima | 2 | 80.0 ± 56.6 |
| methylphenidate | Methylin ER | 1 | 10.0 |
| mirtazapine | Remeron, Remeron SolTab | 5 | 21.0 ± 13.4 |
| nefazodone |  | - | - |
| notriptyline | Aventyl, Pamelor | 2 | 17.5 ± 10.6 |
| olanzapine | Zyprexa, Zyprexa Zydis | - | - |
| paroxetine | Paxil, Paxil CR, Pexeva | 11 | 21.0 ± 11.0 |
| phenelzine | Nardil | - | - |
| protriptyline | Vivactil | - | - |
| quetiapine | Seroquel XR | 4 | 81.3 ± 55.4 |
| selegiline | Emsam | - | - |
| sertraline | Zoloft | 46 | 73.3 ± 32.8 |
| tranylcypromine | Parnate | - | - |
| trazadone | Desyrel, Desyrel Dividose | 42 | 87.2 ± 54.7 |
| trimipramine | Surmontil | - | - |
| venlafaxine | Effexor, Effexor XR | 19 | 96.6 ± 46.2 |
| vilazodone | Viibryd | 4 | 27.5 ± 15.0 |
| vortioxetine | Trintellix | 4 | 15.0 ± 5.8 |

Note: 69 participants were taking more than one antidepressant medication.

**Supplementary Table 3. Sensitivity Analysis Excluding Aripiprazole**

|  |  |  | **CA1** | | **CA23DG** | |
| --- | --- | --- | --- | --- | --- | --- |
| Subgroup | n | Predictor | β | p | β | p |
| Overall | 617 | Medications | -0.12 | 0.08 | -0.11 | 0.09 |

Supplementary Table 3. With 7 participants who were taking aripiprazole excluded, we ran linear regressions between antidepressant medication use and CA1 and CA23DG volumes. Covariates included age, sex, years of education, number of MRI slices segmented, ICV, scanner, BMI, and Rapid Assessment of Physical Activity. All P-values listed are FDR-corrected.

**Supplementary Table 4. Antidepressant Medications by Drug Class**

| **Drug Class** | **Frequency** | **%** |
| --- | --- | --- |
| Atypical Antipsychotic | 11 | 3.2 |
| Central Nervous System (CNS) Stimulant | 1 | 0.3 |
| Miscellaneous | 65 | 19.0 |
| Phenylpiperazine | 42 | 12.3 |
| Serotonin-Norepinephrine Reuptake Inhibitors (SNRI) | 57 | 16.7 |
| Selective-Serotonin Reuptake Inhibitor (SSRI) | 149 | 43.6 |
| Tetracyclic | 5 | 1.5 |
| Tricyclic | 12 | 3.5 |

Supplementary Table 4. The most common antidepressant drug class in our sample is SSRI.

**Supplementary Table 5. GDS Score and Age of Onset of Depression**

|  |  |  | **CA1** | | **CA23DG** | | **Subiculum** | |
| --- | --- | --- | --- | --- | --- | --- | --- | --- |
| Subgroup | n | Predictor | β | p | β | p | β | p |
| Overall | 2009 | Geriatric Depression Scale | -0.01 | 0.5 | -0.03 | 0.28 | 0.01 | 0.6 |
| Overall | 462 | Age of Onset of Depression | -0.05 | 0.25 | -0.001 | 0.98 | -0.06 | 0.25 |

Supplementary Table 5. Separate linear regressions were run to test the effect of GDS score and age of onset of depression on hippocampal subfield volume in the overall sample. Covariates included age, sex, years of education, number of MRI slices segmented, ICV, and scanner. All P-values listed are FDR-corrected.

**Supplementary Table 6. Model 2: Base model with BMI + Rapid Assessment of Physical Activity as additional covariates**

|  |  |  | **CA1** | | **CA23DG** | |
| --- | --- | --- | --- | --- | --- | --- |
| Subgroup | n | Predictor | β | p | β | p |
| Overall | 1992 | Depression | -0.03 | 0.27 | -0.11 | 0.01* |
| *APOE4 -* | 1245 | Depression | -0.06 | 0.24 | -0.14 | 0.01* |
| *APOE4 +* | 437 | Depression | -0.02 | 0.75 | -0.04 | 0.75 |
| Male | 661 | Depression | -0.13 | 0.049* | -0.22 | 0.02* |
| Female | 1331 | Depression | 0.001 | 0.99 | -0.07 | 0.13 |
| Hispanic | 760 | Depression | -0.05 | 0.34 | -0.07 | 0.34 |
| NHB | 385 | Depression | 0.04 | 0.53 | -0.06 | 0.53 |
| NHW | 847 | Depression | -0.06 | 0.37 | -0.1 | 0.25 |
| Depressed | 624 | Medication | -0.12 | 0.06 | -0.12 | 0.07 |

Supplementary Table 6. Separate linear regressions were run to test the effect of depression on hippocampal subfield volume in the overall sample and subset by *APOE*4 status, sex, and ethnoracial group. Additional linear regressions were run to test the effect of depression medication usage on hippocampal subfield volume in depressed participants. Covariates included age, sex, years of education, number of MRI slices segmented, ICV, scanner, BMI, and Rapid Assessment of Physical Activity. All P-values listed are FDR-corrected. P-values that were significant after FDR correction are marked with *. 17 participants were missing either BMI or RAPA data. 310 participants were missing genetic information.

**Supplementary Table 7. Model 2 with ethnoracial group as an additional covariate**

|  |  |  | **CA1** | | **CA23DG** | |
| --- | --- | --- | --- | --- | --- | --- |
| Subgroup | n | Predictor | β | p | β | p |
| Overall | 1992 | Depression | -0.04 | 0.27 | -0.09 | 0.06 |
| *APOE4 -* | 1245 | Depression | -0.04 | 0.39 | -0.12 | 0.04* |
| *APOE4 +* | 437 | Depression | -0.02 | 0.95 | -0.005 | 0.95 |
| Male | 661 | Depression | -0.12 | 0.07 | -0.18 | 0.06 |
| Female | 1331 | Depression | -0.01 | 0.75 | -0.05 | 0.32 |
| Depressed | 624 | Medication | -0.13 | 0.05 | -0.10 | 0.14 |

Supplementary Table 7. Separate linear regressions were run to test the effect of depression on hippocampal subfield volume in the overall sample and subset by *APOE*4 status and sex. Additional linear regressions were run to test the effect of depression medication usage on hippocampal subfield volume in depressed participants. Covariates included age, sex, years of education, number of MRI slices segmented, ICV, scanner, BMI, Rapid Assessment of Physical Activity, and ethnoracial group. All P-values listed are FDR-corrected. P-values that were significant after FDR correction are marked with *. 17 participants were missing either BMI or RAPA data. 310 participants were missing genetic information.

**Supplementary Table 8. GDS Scores and History of Depression**

|  | **GDS≥10** | **GDS<10 + History of depression** |
| --- | --- | --- |
| N | 343 | 287 |
| Self-reported history of depression: n (%)  Yes  No | 178 (51.9%)  165 (48.1%) | 287 (100.0%)  0 (0.0%) |
| Medication use: n (%)  Medicated  Not medicated | 102 (29.7%)  241 (70.3%) | 161 (56.1%)  126 (43.9%) |
| Chi-square test for medication use | t= 44.65  p**< 0.001*** | |
| Association with CA23DG volume | β= 0.002  p= 0.97 | β= -0.17  p= **0.06** |
| Association with medication use | β= -0.02  p= 0.84 | β= -0.21  p= **0.02*** |

Supplementary Table 8. Breakdown of self-reported history of depression and medication status of participants grouped by GDS≥10 (regardless of depression history) and GDS<10 with a depression history. Linear regression models were run to test the association between GDS score and medication use with CA23DG volume in each group. Covariates included age, sex, years of education, BMI, RAPA, number of slices segmented, ICV, and scanner. * indicates p<0.05.

**Supplementary Table 9. Base Linear Regression Model- Stratified**

|  |  |  | **CA1** | | **CA23DG** | | **Subiculum** | |
| --- | --- | --- | --- | --- | --- | --- | --- | --- |
| Subgroup | n | Predictor | β | p | β | p | β | p |
| *APOE*4 - | 1258 | Depression | -0.07 | 0.14 | -0.14 | 0.01* | 0.03 | 0.49 |
| *APOE*4 + | 439 | Depression | -0.03 | 0.64 | -0.04 | 0.64 | 0.07 | 0.64 |
| Male | 668 | Depression | -0.16 | 0.02* | -0.23 | 0.01* | -0.03 | 0.65 |
| Female | 1341 | Depression | -0.01 | 0.84 | -0.08 | 0.10 | 0.10 | 0.05 |
| Hispanic | 763 | Depression | -0.06 | 0.27 | -0.08 | 0.27 | 0.06 | 0.27 |
| NHB | 388 | Depression | 0.04 | 0.52 | -0.07 | 0.52 | 0.12 | 0.42 |
| NHW | 858 | Depression | -0.08 | 0.13 | -0.12 | 0.12 | -0.05 | 0.41 |

Supplementary Table 9. Separate linear regressions were run to test the effect of depression on hippocampal subfield volume in subgroups. Covariates included age, sex, years of education, number of MRI slices segmented, ICV, and scanner. All P-values listed are FDR-corrected. NHB=non-Hispanic Black, NHW=non-Hispanic White. * indicates p<0.05. 312 participants are missing *APOE*4 genotype data.

**Supplementary Table 10. Interactions by Global Amyloid and MTL Tau PET SUVRs**

|  | **CA1** | | **CA23DG** | | **Subiculum** | |
| --- | --- | --- | --- | --- | --- | --- |
|  | β | p | β | p | β | p |
| Global amyloid PET | -0.08 | 0.37 | -0.04 | 0.67 | -0.01 | 0.88 |
| MTL tau PET | 0.01 | 0.91 | -0.04 | 0.91 | 0.02 | 0.91 |

Supplementary Table 10. Formal interactions were run for global amyloid and MTL tau PET SUVRs and depression on CA1, CA23DG, and subiculum volumes. Covariates included age, sex, years of education, number of MRI slices segmented, ICV, scanner, BMI, and RAPA. All P-values listed are FDR-corrected. MTL= medial temporal lobe.

**Supplementary Table 11. Depression and *APOE*4 Positivity**

|  |  |  | **Depression** | |
| --- | --- | --- | --- | --- |
| Subgroup | n | Predictor | β | p |
| Overall | 1682 | *APOE*4 Positivity | 0.01 | 0.95 |

Supplementary Table 11. A linear regression was run to test the effect of *APOE*4 Positivity on depression in the overall sample. Covariates included age, sex, years of education, number of MRI slices segmented, ICV, scanner, BMI, and Rapid Assessment of Physical Activity.
